# Biotechnological Potential of Algerian Saffron Floral Residues: Recycling Phytochemicals with Antimicrobial Activity

**DOI:** 10.3390/biology15020197

**Published:** 2026-01-21

**Authors:** Nouria Meliani, Bouchra Loukidi, Larbi Belyagoubi, Nabila Belyagoubi-Benhammou, Salim Habi, Alessia D’Agostino, Antonella Canini, Saber Nahdi, Nassima Mokhtari Soulimane, Angelo Gismondi, Abdel Halim Harrath, Erdi Can Aytar, Gabriele Di Marco

**Affiliations:** 1 Laboratory of Physiology, Physiopathology and Biochemistry of Nutrition, Department of Biology, Faculty of Natural and Life Sciences, Earth and Universe Sciences, University Abou-Bekr Belkaïd, Tlemcen 13000, Algeria; noriameliani@gmail.com (N.M.); loukbou21@gmail.com (B.L.); salim.habi@yahoo.com (S.H.); nassima_amel@yahoo.fr (N.M.S.); 2 Laboratory of Natural Products, Department of Biology, Faculty of Natural and Life Sciences, Earth and Universe Sciences, University Abou-Bekr Belkaïd, Tlemcen 13000, Algeria; belyagoubi_larbi@yahoo.fr (L.B.); nabila.benhammou79@yahoo.fr (N.B.-B.); 3 Department of Biology, University of Rome Tor Vergata, Via della Ricerca Scientifica 1, 00133 Rome, Italy; d.agostino@scienze.uniroma2.it (A.D.); canini@uniroma2.it (A.C.); gismondi@scienze.uniroma2.it (A.G.); 4 Department of Zoology, College of Sciences, King Saud University, Riyadh 11451, Saudi Arabia; nehdisabeur@gmail.com (S.N.); hharrath@ksu.edu.sa (A.H.H.); 5 Faculty of Agriculture, Department of Horticulture, Usak University, 64200 Usak, Turkey; erdicanaytar@gmail.com

**Keywords:** saffron flower waste, HPLC-DAD, polyphenols, antioxidant effect, antimicrobial activity, molecular docking

## Abstract

This study investigates the phytochemical profile, antioxidant capacity, and antimicrobial potential of *Crocus sativus* L. (saffron) tepal extracts obtained via different solvent systems. Here, a biochemical screening was performed using spectrophotometry and HPLC-DAD, while molecular docking simulations were carried out to evaluate the possible interactions between saffron tepal metabolites and bacterial target proteins. In parallel, antioxidant activity was assessed using radical scavenging assays, whereas antimicrobial potential (i.e., MIC, MBC, and MFC) was tested against selected bacterial strains. Results indicated that aqueous successive and crude extracts yielded the highest concentrations of polyphenols, flavonoids, and condensed tannins. In detail, HPLC-DAD analysis specifically identified significant levels of gallic acid, epicatechin, and various anthocyanins. These extracts demonstrated robust antioxidant and antimicrobial activities. This latter evidence was corroborated by the docking analyses, which revealed that chlorogenic acid and petunidin-3-glucoside exhibited high binding affinities for 2NRK and 2NZF, whereas epicatechin and pelargonidin effectively targeted 8ACR. These findings underscore the therapeutic potential of *C. sativus* tepals as natural bioactive agents, suggesting a promising role in overcoming antibiotic resistance and supporting their development for pharmaceutical applications.

## Simple Summary

This study explores the bioactive compounds found in *Crocus sativus* L. tepals, evaluating their antimicrobial and antioxidant potential. Using advanced extraction techniques, we identified gallic acid, epicatechin, and chlorogenic acid as primary constituents. The resulting extracts demonstrated a robust capacity for neutralizing free radicals and inhibiting bacterial growth. Furthermore, computational simulations suggest that these compounds effectively interact with bacterial proteins, potentially disrupting their function. These findings highlight *C. sativus* tepals as promising natural health-promoting agents and a valuable resource for developing novel antibacterial therapies.

## 1. Introduction

Phytotherapy is a cornerstone of complementary and alternative medicine, playing a vital role in disease prevention and treatment, particularly within non-Western societies. Recently, the scientific validation of traditional ethnobotanical practices has spurred a renewed interest among researchers and the pharmaceutical industry in the development of herbal-based drugs [1,2]. Among medicinal plants, saffron stands out as a preeminent example, with a therapeutic legacy deeply rooted in diverse traditional and cultural practices.

*Crocus sativus* L. is a perennial, geophytic, autumn-flowering herb belonging to the Iridaceae family. It is a Mediterranean species widely cultivated in several countries, such as Spain, Greece, Italy, Turkey, and Algeria, as well as Iran, India, Morocco, Azerbaijan, and China [3]. Due to its medicinal properties, the use of saffron is continuously increasing. Extensive research has documented its efficacy across a broad spectrum of health disorders, prompting farmers to introduce *C. sativus* as an allochthonous species and adopt innovative cultivation practices in new regions to satisfy rising global demand [4,5].

The chemical profile of saffron comprises approximately 150 volatile and non-volatile compounds. Among the volatile constituents—primarily terpenoids and terpene alcohols—safranal stands out as the most biologically active [6]. Conversely, the non-volatile fraction includes metabolites such as picrocrocin and crocins; these are responsible for saffron’s characteristic bitterness, intense coloration and distinct aroma, respectively [6,7]. Bioactive metabolites are distributed throughout the plant—including the stigmas, tepals, leaves, and corms—albeit in varying concentrations. These secondary metabolites exert their therapeutic effects by modulating a wide range of physiological and biological pathways [8].

Numerous studies have reported the beneficial effects of saffron phytochemicals on several pathological conditions, including cancer, diabetes, cardiovascular diseases, erectile dysfunction, and Alzheimer’s disease [9]. Experimental and animal studies have further demonstrated the antioxidant, antimicrobial, hepatoprotective, and antineoplastic activities of saffron, supporting its use as a nutraceutical and functional food ingredient [10,11,12]. In addition to its bioactive properties, saffron contains essential nutrients, such as proteins, fibers, carbohydrates, and minerals, whose concentrations vary depending on cultivation conditions, soil fertility, and geographical origin [13,14,15]. Saffron stigmas are also rich in minerals, amino acids and vitamins, which contribute to their nutritional and medicinal value [16,17,18,19,20]. However, *C. sativus* stamens and tepals are also rich in fatty acids [21,22].

During saffron processing, only the stigmas are harvested for commercial use, leaving the remaining floral organs—particularly the tepals—to be discarded as agricultural waste. It is estimated that approximately 350 kg of tepals are generated and remain unutilized per production cycle [15,23,24]. Despite being classified as a bioresidue, saffron tepals possess lower lipid and protein content but exhibit significantly higher levels of minerals and carbohydrates compared to the stigmas [25,26]. Tepals are also recognized for their significant antioxidant activity, which is attributed to the presence of carotenoids, phenolic compounds, flavonoids, and anthocyanins [25,27,28,29,30]. These phytochemicals play a crucial role in human nutrition by mitigating oxidative stress and promoting overall physiological health [31].

In this context, the present study aims to valorize *C. sativus* flower residues collected from Djebel Zaafran in Ain Fezza (Tlemcen Province, western Algeria). The tepals underwent maceration in water and various solvents, following a gradient of increasing polarity. Antioxidant activity and antibacterial properties of the various extracts were evaluated, together with their chemical composition, via high-performance liquid chromatography associated with diode array detection (HPLC-DAD) and spectrophotometry. Lastly, molecular docking simulations were performed to examine the potential interactions between the major phytochemicals identified in saffron tepals and selected bacterial proteins, namely, 8ACR (aminopeptidase from *Pseudomonas aeruginosa*), 2NRK (conserved protein GrpB from *Enterococcus faecalis*), 2NZF (beta-lactamase II from *Bacillus cereus* R121H, C221S double mutant), and 3QNE (seryl-tRNA synthetase from *Candida albicans*), in order to justify the antimicrobial potential of the investigated plant metabolites.

## 2. Experimental

### 2.1. Chemicals and Reagents

Dichloromethane, Ethyl acetate, Acetone, n-Butanol, Methanol, formic acid, dimethyl sulfoxide (DMSO), gallic acid, catechin, quercetin, Folin-Ciocalteu, 2,2-diphenyl-1-picrylhydrazyl (DPPH), ascorbic acid, Muller-Hilton agar, Muller-Hilton broth, Sabouraud dextrose agar, Sabouraud broth and Nystatin were purchased from Sigma (St. Louis, MO, USA); Ampicillin was provided by Biocare (Algiers, Algeria). All remaining chemicals used were of analytical grade and were purchased from Sigma.

### 2.2. Material

The flowers of *C. sativus* were collected from Djebel Zaafran of Ain Fezza Commune (latitude: 34°52′36″ N; longitude: 01°12′55″ W; altitude: 863 m; Tlemcen Province; Algeria) between the 15th of October and the 15th of November 2021. Identification of the plants was performed by Prof. F. Hassani (Laboratory of Ecology and Management of Natural Ecosystems, University of Tlemcen, Algeria). A voucher specimen has been deposited at the Laboratory of Natural Products (Department of Biology, University of Tlemcen) under the accession *Crocus* L. 224. In the laboratory, the tepals were dried at room temperature and in the shade, ground into a fine powder, and stored at 4 °C.

### 2.3. Extraction Procedure

First, tepal powder was subjected to lipid extraction by resuspension in 500 mL of hexane using a Soxhlet apparatus for four hours. The recovered pellet was completely dried, weighed, and macerated with 350 mL of dichloromethane for 48 h at room temperature. The resulting solution was then filtered through Whatman filter paper (0.8 µm porosity). The dichloromethane extract was obtained after the complete evaporation of the solvent using a rotary evaporator and resuspending the residue with DMSO at the indicated concentrations.

The remaining solid residue was weighed and macerated in 350 mL of ethyl acetate to obtain, after solvent evaporation, the ethyl acetate extract. The same procedure described for the dichloromethane extract was applied, and the residual pellet was subsequently extracted using other solvents, including acetone, *n*-butanol, methanol, and distilled water. After filtration and evaporation, the ethyl acetate and acetone extracts were dissolved in DMSO, as for the dichloromethane extract, whereas the *n*-butanol extract was dissolved in methanol. In contrast, the methanolic and aqueous extracts were maintained in their respective solvents. The solvent successive extracts obtained were designated as dichloromethane successive extract (DSE), ethyl acetate successive extract (EASE), acetone successive extract (AcSE), *n*-butanol successive extract (BSE), methanol successive extract (MSE), and aqueous successive extract (ASE). All extracts were stored at 4 °C in the dark until analysis.

In parallel, an aqueous crude extract (ACE) was prepared by directly macerating the delipidated tepal powder pellet in 500 mL of distilled water at 6 °C for 48 h. After filtration, the solution was evaporated using a rotary evaporator to obtain the aqueous crude extract, which was reconstituted in distilled water and stored at 4 °C in the dark.

### 2.4. Determination of the Total Content of Secondary Metabolites

The Folin-Ciocalteu method [32] was employed to determine the total phenolic content. Results were expressed as milligrams of gallic acid equivalent (GAE) per gram of dry matter (DM), using a calibration curve established with increasing concentrations of pure gallic acid (R^2^ = 0.9909). To assess total flavonoids, the aluminum chloride colorimetric test was conducted according to Zhishen et al. [33], with results reported as milligrams of catechin equivalent (CE) per gram of dry matter (DM) based on a catechin standard curve (R^2^ = 0.9984).

The concentration of condensed tannins in the samples was measured using the vanillin assay described by Julkunen-Tiitto [34]. Total condensed tannin content was represented as milligrams of catechin equivalent (CE) per gram of dry matter (DM), using a calibration curve derived from various concentrations of catechin standard (R^2^ = 0.9983). Hydrolysable tannins were assessed following the method of Mole and Waterman [35]. The results were expressed in milligrams of gallic acid equivalent (GAE) per gram of dry matter (DM) using a calibration curve based on gallic acid. Finally, flavonol concentration was determined using the method outlined by Kumaran and Karunakaran [36]. The quantification based on a quercetin standard curve was expressed as milligrams of quercetin equivalent per gram of dry matter (mg QE/g DM).

### 2.5. HPLC-DAD Analysis

The aqueous successive extract and the aqueous crude extract of saffron were completely dried and subsequently resuspended in 5 mL of extraction solvent (MeOH:H_2_O, 50:50, *v*/*v*) for 24 h with continuous agitation.

The biochemical profiles of both samples were analyzed using a high-performance liquid chromatography (HPLC) system, which included a CBM-20A controller, an LC-20 AD pump, an SIL-20A HT autosampler, and an SPD-M20A diode array detector (DAD) (Shimadzu, Kyoto, Japan), as reported in Belarbi et al., 2025 [37]. Chromatographic separation was achieved using a Luna 3u C18(2) column (150 mm × 4.60 mm × 3 μm) (Phenomenex, Torrance, CA, USA), with mobile phases comprising 1% formic acid (*v*/*v*) (phase A) and MeOH (phase B) at a flow rate of 0.95 mL/min. The elution commenced with 15% B solvent, maintained for 20 min, then linearly increased to 35% B over 20 min and up to 90% in 55 min. At 70 min, the flow returned to the initial condition of 15% B. Twenty microliters of each sample were injected into the HPLC system, with the column temperature set at 40 °C. Data acquisition was conducted using LAB-SOLUTION software (v-2010, Shimadzu). Phenolic compounds (including gallic acid, vanillic acid, rosmarinic acid, chlorogenic acid, caffeic acid, syringic acid, ρ-coumaric acid, and salicylic acid) and flavonoids (such as resveratrol, myricetin, quercetin, kaempferol, and epicatechin) were quantified and monitored at 280 nm. Qualitative identification and quantitative analysis of each plant compound were carried out by comparing their retention times, absorption spectra, and peak areas to those of pure standard molecules (Sigma–Aldrich, Milan, Italy). Results are expressed as nanograms of standard equivalent per milligram of sample dry weight.

### 2.6. Quantitation of Specific Anthocyanins

One gram of tepal powder was resuspended in 10 mL of extraction solvent (MeOH:H_2_O, 50:50; *v*/*v*) at room temperature and kept in the dark for 24 h with agitation. Following centrifugation at 6000× *g* for 10 min, two buffers were prepared: a 0.025 M potassium chloride buffer at pH 1.0 and a 0.4 M sodium acetate buffer at pH 4.5, as previously described by Giusti and Wrolstad [38]. Next, 50 µL of each sample was mixed separately with 50 µL of the corresponding buffer solution. These mixtures were incubated in the dark for 15 min, after which absorbance was measured at various wavelengths: 510 nm for cyanidin, 523 nm for delphinidin, 557 nm for malvidin, 505 nm for pelargonidin, 496 nm for pelargonidin 3-glucoside, 511 nm for peonidin, 520 nm for petunidin 3-glucoside, and 700 nm as a background.

To calculate the absorbance of the samples, the following formula was applied:

A = [(A_x_ − A_700_)pH_1.0_ − (A_x_ − A_700_)pH_4.5_]A = [(A_x_ − A_700_)pH_1.0_ − (A_x_ − A_700_)pH_4.5_]

where x represents the wavelength at which each anthocyanin absorbs.

The concentration of each anthocyanin was determined using the equation:

Concentration = (A × MW × dilution factor × 1000) (ε × l)

Here, A is the measured absorbance for each anthocyanin; MW is the molecular weight of the selected anthocyanin (cyanidin: 287.24; delphinidin: 303.24; malvidin: 331.29; pelargonidin: 271.24; pelargonidin 3-glucoside: 433.4; peonidin: 301.27; petunidin 3-glucoside: 479.41); ε is the molar absorptivity (cyanidin: 24,600; delphinidin: 34,700; malvidin: 36,200; pelargonidin: 17,800; pelargonidin 3-glucoside: 15,600; peonidin: 37,200; petunidin 3-glucoside: 18,900); l is the path length of the cuvette. The results are expressed as micrograms of anthocyanin per milligram of dry weight (µg/mg DW).

### 2.7. Evaluation of the Antioxidant Activity

The ability of the plant extracts to donate hydrogen atoms was assessed based on their capacity to scavenge the 2,2-diphenyl-1-picrylhydrazyl (DPPH) free radical [39]. Fifty microliters of various concentrations of different plant samples were mixed with 1950 μL of a 0.025 g/L DPPH methanolic solution. After incubating for 30 min at room temperature, the absorbance was measured at 515 nm against a blank containing all reagents except the plant extract. The DPPH free radical scavenging activity was calculated as a percentage using the following formula:

DPPH scavenging activity (%) = (A_blank_ − A_sample_)/A_blank_ × 100

where A_blank_ represents the absorbance of the control reaction (the blank), and A_sample_ is the absorbance of the solution containing the extract. The concentration of the sample that inhibited the DPPH radical solution by 50% (i.e., EC_50_) was extrapolated, and the results of this test are reported. Ascorbic acid served as a positive control.

The total antioxidant capacity (TAC) of the plant extracts was assessed using the phosphomolybdenum method [40]. An aliquot of 0.3 mL of the sample was combined with 3 mL of a standard reagent consisting of 0.6 M sulfuric acid, 4 mM ammonium molybdate, and 28 mM sodium phosphate. The mixture was incubated for 90 min at 95 °C. Afterward, the reaction mixture was cooled to room temperature, and absorbance was measured at 695 nm. The TAC was expressed as milligrams of ascorbic acid equivalents (AAE) per gram of dry matter (DM).

### 2.8. Evaluation of Antimicrobial Activity

The antibiotic effects of the extracts were tested against four bacterial species: the Gram-positive bacteria *Enterococcus faecalis* (ATCC 29212) and *Bacillus cereus* (ATCC 11778), as well as the Gram-negative bacteria *Escherichia coli* (ATCC 25922) and *Pseudomonas aeruginosa* (ATCC 27853). Additionally, two yeast strains, *Candida albicans* (CIP 444) and *Candida albicans* (ATCC 10231), were included in the study. The microbial stocks were revived, and their turbidity was adjusted to 0.5 McFarland, corresponding to approximately 10^8^ CFU/mL for bacteria (O.D. = 0.08 to 0.1 at λ = 625 nm) and approximately 10^6^ CFU/mL for yeast (O.D. = 0.12 to 0.15 at λ = 530 nm) [41]. Inhibition zones were assessed using the disc diffusion method, following Clinical and Laboratory Standards Institute (CLSI) guidelines [42,43]. Bacterial suspensions were prepared in Mueller–Hinton broth (MHB), while yeast suspensions were prepared in Sabouraud dextrose broth (SDB). The cultures were incubated at 37 °C for 24 h for bacteria and at 30 °C for 24 to 48 h for yeast. Once the appropriate concentrations were achieved, each culture suspension was spread onto solid media plates using a sterile cotton swab. Whatman sterile filter paper discs (6 mm in diameter) were impregnated with 7.5/10 µL of the various plant extracts and placed in the center of the plates. Positive controls included ampicillin (10 μg; Biocare Algeri, Algeria) for bacteria and nystatin (100 μg; Sigma, Aizu, Fukushima, Japan) for yeast, used to validate the sensitivity of the tested microorganisms. The plates were left for 2 h at 4 °C and then incubated for 24 h at 37 °C for bacteria and 48 h at 30 °C for yeast. The diameters of the inhibition zones (IZDs), including those of the paper discs, were measured in millimeters (mm) [44].

The minimum inhibitory concentrations (MICs) of the plant extracts were determined using the micro broth dilution method [45,46], in accordance with CLSI protocols. All tests were conducted in Mueller–Hinton broth (Sabouraud dextrose broth for *C. albicans*), and the cultures of each strain were prepared overnight. Microorganism suspensions were adjusted to a final density of 10^6^ CFU/mL using a spectrophotometer (Cary 50 Bio UV-Visible, Varian, Agilent Technologies, Torino, Italy). One hundred microliters of each sample were serially diluted twofold in 96-well microtiter plates, combined with equal volumes of Mueller–Hinton broth (or Sabouraud dextrose broth for *C. albicans*). Subsequently, 100 μL of inoculum was added to each well, resulting in a final concentration range of 5–0.019 mg/mL. After incubation for 24 h at 37 °C (or 48 h for yeast) in a normal atmosphere, the MIC was determined. This is defined as the lowest concentration of the extract that inhibits visible growth of the tested microorganism, indicated by turbidity in the medium. The minimum bactericidal concentration (MBC) was assessed by subculturing 10 μL from wells showing no visible bacterial growth onto Mueller–Hinton agar (MHA) (or Sabouraud dextrose agar for *C. albicans*), followed by incubation at 37 °C for 24 h (or 48 h for *Candida*) [27]. The MIC and MBC results are expressed in mg/mL.

### 2.9. Computational Details

In this study, the antimicrobial effects of different extracts obtained from *C. sativus* tepals were evaluated. Based on inhibition zone diameters, minimum inhibitory concentration (MIC), and minimum bactericidal/fungicidal concentration (MBC/MFC) analyses, bacterial and fungal strains exhibiting high susceptibility to the tested extracts were selected. In our study, the crystal structures of 8ACR (*P. aeruginosa* aminopeptidase), 2NRK (*E. faecalis* GrpB protein), 2NZF (*B. cereus* beta-lactamase II), and 3QNE (*C. albicans* Ser-yl-tRNA synthetase) were selected as model targets due to their relevance to the experimental antimicrobial results and their structural suitability for molecular docking analysis. In particular, *P. aeruginosa* and *E. faecalis* showed the highest sensitivity in our study based on disk diffusion, MIC, and MBC analyses. Therefore, experimentally resolved protein structures from these organisms were chosen as model targets to evaluate the potential molecular interactions of plant-derived compounds. Accordingly, *P. aeruginosa, E. faecalis, B. cereus*, and *C. albicans* CIP were included in further ADMET and molecular docking analyses due to their high susceptibility to the extracts. In contrast, *E. coli* and *C. albicans* ATCC were excluded from the study due to their low susceptibility to the tested extracts. Furthermore, the selection of bioactive compounds for ADMET and molecular docking analyses was based on the highest concentrations and reported significant bioactivity of specific phytochemicals in prior research. Therefore, gallic acid, epicatechin, 4-hydroxybenzoic acid, chlorogenic acid, resveratrol, pelargonidin, pelargonidin 3-glucoside, and petunidin 3-glucoside were prioritized for further investigation. This approach allowed for a detailed evaluation of the bioavailability and interaction potential of the most bioactive phytochemicals with target proteins in the selected microorganisms.

Molecular docking simulations were conducted to investigate the interactions between the major compounds identified through HPLC-DAD analysis and anthocyanin quantification with selected target proteins, including 8ACR (*P. aeruginosa* aminopeptidase), 2NRK (conserved protein GrpB from *E. faecalis*), 2NZF (beta-lactamase II from *B. cereus* R121H, C221S double mutant in space group C2), and 3QNE (*C. albicans* seryl-tRNA synthetase). Before docking, all water molecules and cofactors were removed to avoid interference in the binding interactions, and polar hydrogen atoms were added using AutoDockTools (ADT) (version 1.5.6) to optimize the protein structures. The ligand structures were retrieved from the PubChem database in SDF format and converted into PDB format using Discovery Studio Visualizer for compatibility with docking simulations. The active site was defined using AutoGrid, with the grid centred on the binding pocket and set to 40 points in each direction, with a grid spacing of 0.375 Å. Docking simulations were carried out using AutoDock Vina, where ten distinct binding conformations were generated for each ligand to assess their affinity toward the target proteins. The energy range was set at 9 kcal/mol, and the exhaustiveness parameter was adjusted to 1000 to ensure the accuracy and reliability of the results. The docking analysis was performed based on key parameters, including binding energy, ligand efficiency (LE), fit quality (FQ), pIC50, and the estimated inhibition constant (Ki). The obtained binding conformations and molecular interactions were visualized in both 2D and 3D representations using BIOVIA Discovery Studio Visualizer [47], allowing for a comprehensive evaluation of ligand-protein interactions. The results of this study provide significant insights into the binding potential and pharmacological relevance of the analyzed compounds against the selected bacterial and fungal target proteins.

The pharmacokinetic properties and safety profiles of the selected phytocompounds were evaluated through ADMET (Absorption, Distribution, Metabolism, Excretion, and Toxicity) analysis and toxicity predictions. A total of seventeen phytocompounds were converted into SMILES notation and processed using an online ADME prediction platform. To predict key pharmacokinetic parameters, including absorption, metabolism, and excretion, as well as fundamental physicochemical characteristics, the SWISSADME tool was employed. These parameters are crucial in assessing the drug-likeness and clinical potential of the compounds studied, providing insight into their suitability for further pharmacological development [48,49].

### 2.10. Statistical Analysis

Three replicas of each experiment were used simultaneously. The outcomes are displayed as the mean ± standard deviation (SD). The obtained data were subjected to one-way analysis of variance (ANOVA) with Tukey’s test using the statistical software IBM SPSS V26. A value of *p* < 0.001 was considered to indicate statistical significance. To interpret the underlying interrelationships between the phytochemical contents of the plant samples and their antioxidant, antibacterial, and antifungal activities, principal component analysis (PCA) was performed using R software for statistical analysis, computing and graphics version 4.2.3.

## 3. Results and Discussion

### 3.1. Total Content of Secondary Metabolites

The yields and content of total phenolics, total flavonoids, condensed tannins, hydrolysable tannins, and total flavonols of the solvent successive extracts (ASE) and the aqueous crude extract (ACE) obtained from *C. sativus* tepals are presented in Table 1. The yield of extracts ranged from 0.901 to 28.394%, with the MSE showing the highest value (28.394%), followed by ASE (26.455%). While ACE showed the greatest yield (62.433%). Similar results have been obtained by Lachguer et al. [50], with saffron produced in southern Morocco. According to their analysis, the yield ranking placed the aqueous crude extract at the top (66.3%), followed by the methanol (49.06%), water (27.87%), n-butanol (10.97%), diethyl ether (1.3%), and ethyl acetate (1.22%) fractions. All the extracts were rich in secondary metabolites, except for dichloromethane. High concentrations of total phenolics, flavonoids and condensed tannins were found in ACE and ASE. The MSE appeared to possess the highest content of total flavonols (57.823 ± 1.478 mg QE/g DM) and hydrolysable tannins (250.733 ± 3.321 mg GAE/g DM). Overall, the phytochemical contents in the floral samples generally declined in the following order: ACE, followed by ASE, MSE, BSE, AcSE, EASE, and DSE.

**Figure 1:**
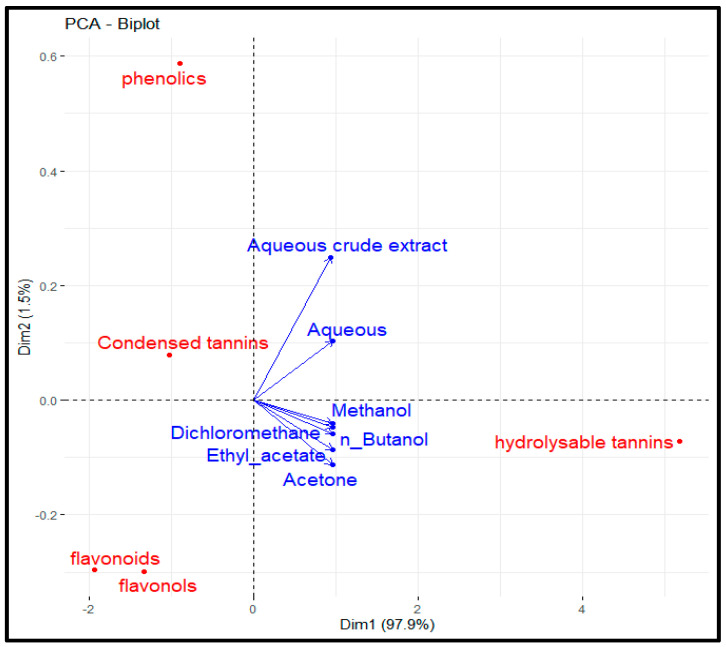
The Principal Component Analysis (PCA) between solvent successive extracts, aqueous crude extract, and phytochemical content.

Principal component analysis (PCA) confirmed these findings (Figure 1). These results were not consistent with those reported for a similar investigation performed on saffron cultivated in Morocco; in that case, the richest phenolic fractions were the diethyl ether (214.29 mg GAE/g DM), n-butanol (171.8 mg GAE/g DM), and ethyl acetate (154.32 mg GAE/g DM) fractions, followed by the aqueous (130.39 mg GAE/g DM) and methanolic (114.66 mg GAE/g DM) fractions and the aqueous crude extract (104.82 mg GAE/g DM) [49]. The flavonoid content detected in the present study was lower than that detected for Moroccan saffron (60.64 ± 2.71 mg CE/g DM), whereas the total phenolic content was similar (65.34 ± 1.74 mg GAE/g DM) [17]. In addition, the results for total phenolics and hydrolysable and condensed tannins were generally in line with those reported for *C. sativus* samples from Algeria (Tlemcen Province; 69.187 mg GAE/g DM, 277.304 ± 6.756 mg GAE/g DM, and 48.854 mg CE/g DM, respectively) [27]. Notably, the presented results were greater than those obtained for saffron cultivated in different areas: the northwestern Italian Alps (total phenolics ranging between 888.35 and 3642.95 mg GAE/100 g DW) [51] and Iran (1.96 ± 0.12 g GAE/100 g DW) [52]. This high variability could be attributed to differences in origins, altitudes, growing conditions, and picking periods. In addition, other parameters that can also be considered possible causes of these fluctuations are environmental conditions, genetic factors, storage, sample preparation techniques, etc. [53]. The richness in tannins and anthocyanins documented for tepals is in line with the data reported by Babaei et al. [54].

### 3.2. Quantitation of Specific Secondary Metabolites

The spectrophotometric analyses of the extracts from *C. sativus* tepals revealed that ASE and ACE were rich in secondary metabolites. They were then subjected to HPLC-DAD to determine their chemical composition, using authentic standards of phenolic compounds as references. In detail, 18 out of the 21 phytochemicals investigated were identified in ACE, and 17 in ASE. The concentrations of these substances in the samples are shown in Table 2.

In the aqueous successive extract, the most abundant compound was 4-hydroxybenzoic acid (9.6210 ± 0.4811 ng/mg), followed by gallic acid (5.1878 ± 0.3113 ng/mg), epicatechin (3.6241 ± 0.1449 ng/mg), chlorogenic acid (3.3271 ± 0.2329 ng/mg), and resveratrol (2.6549 ± 0.1062 ng/mg). The same molecules (even in the same order of concentration) were found in the aqueous crude extract. These results are lower than those reported by Belyagoubi et al. [27]. These authors showed the presence of 4-hydroxybenzoic acid (0.304 ± 0.022 μg/mg), gallic acid (1.087 ± 0.048 μg/mg), epicatechin (0.128 ± 0.065 μg/mg), chlorogenic acid (0.119 ± 0.018 μg/mg), and resveratrol (0.029 ± 0.018 μg/mg). This difference could be due to the different environmental conditions present in the cultivation area or to the extraction method applied to the plant material. Another study focusing on the phytocomplex of different parts of the saffron flower revealed the dominance of flavonols, such as quercetin and methyl-quercetin glycosides, in the tepals and kaempferol derivatives in the stamens [55]. Moreover, the kaempferol, quercetin, naringenin, and some glycosylated flavanones and flavanols esterified with phenylpropanoic acids were particularly abundant in *C. sativus* tepals [56]. In addition, kaempferol-3-O-β-sophoroside [24,57] and quercetin-3-O-sophoroside [58] have been demonstrated to be the major flavonoid present in saffron flowers [24,57]. Ruggieri et al. [59] have found in saffron tepals a high concentration of flavonoids, including kaempferol glycosides, quercetin, and isorhamnetin glycosides, known for their antioxidant and anti-inflammatory properties. In our study, both quercetin derivatives and kaempferol were detected in the aqueous saffron extracts, although at low doses, whereas vanillic acid was present only in the crude extract. About the anthocyanins, the presence of seven compounds was revealed (Table 3). Petunidin 3-glucoside was the most abundant compound (3.890 ± 0.233 µg/mg), whereas peonidin was the least abundant (1.127 ± 0.068 µg/mg). These results were generally higher than those reported by Belyagoubi et al. [27] for samples collected at the same station (cyanidin, 0.024 ± 0.015 μg/mg; petunidin, 0.035 ± 0.018 μg/mg; peonidin, 0.019 ± 0.002 μg/mg; and malvidin, 0.034 ± 0.020 μg/mg), suggesting that the environment plays a key role in shaping the phytocomplex of this species. Other scholars have reported that saffron tepals are characterized by high levels of anthocyanins [24,54]. This family contributes to their color and potential health benefits [60], with reported levels reaching up to 413.30 mg cyanidin 3-O-glucoside per 100 g dry weight [61]. Other compounds registered in this plant material are carotenoids, monoterpenoids, and vitamins, such as Vitamin C, enhancing the nutritional profile of saffron tepals [61,62].

### 3.3. Antioxidant Activity

**Figure 2:**
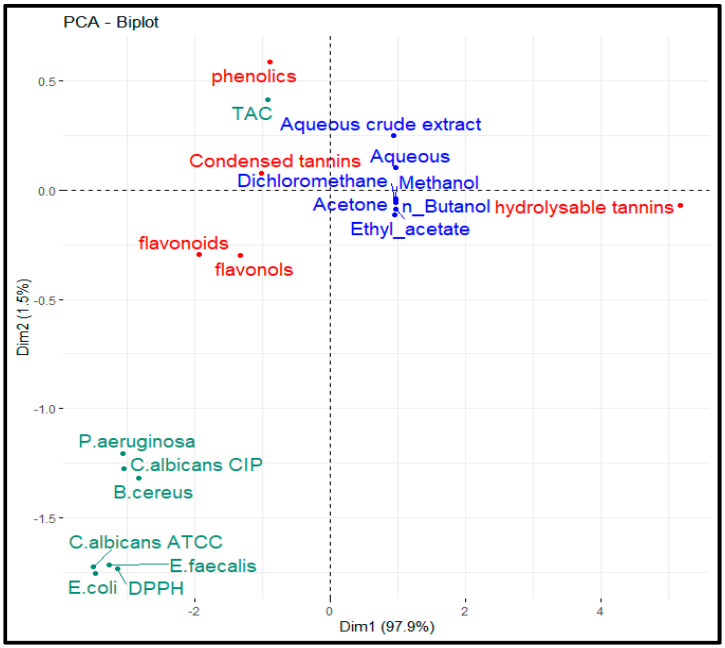
PCA of the correlations between phytochemical contents, total antioxidant activity (TAC), DPPH radical-scavenging power, antibacterial and antifungal activities.

Total antioxidant capacity (TAC) and DPPH radical scavenging tests were conducted to assess the antioxidant capability of the flower residues of aqueous crude extract and solvent successive extracts; the findings are reported in Table 4. All the extracts showed good TAC values, except the dichloromethane extract. In particular, as expected on the basis of the previous analyses (i.e., content of secondary metabolites), methanolic and aqueous preparations were the most bioactive. PCA revealed a strong correlation between phenolic content and TAC (Figure 2). Similar results were obtained for the DPPH assay. In this case, the antioxidant effect of the plant extracts on the DPPH radical is due to their hydrogen-donating ability, which reduces the stable purple DPPH free radical to a yellow nonradical DPPH-H form [63]. Here, ascorbic acid, which was used as a positive control, presented a very high radical scavenging capacity (0.060 ± 0.001 mg/mL). Among the samples, the ASE had the greatest DPPH reducing potential (EC_50_ = 2.646 ± 0.09 mg/mL), followed by the ACE (2.669 ± 0.463 mg/mL), methanol, butanol, acetone and ethyl acetate extracts. These results were not consistent with those from Lachguer et al. [50], indicating that diethyl ether extract from *C. sativus* tepals had greater antioxidant effects (EC_50_ = 309.44 µg/mL) than ethyl acetate (EC_50_ = 721.09 µg/mL), n-butanol (EC_50_ = 883.96 µg/mL), aqueous (EC_50_ = 888.77 µg/mL), methanol (EC_50_ = 1364.8 μg/mL), and aqueous crude extracts (EC_50_ = 1226.98 µg/mL). However, Jadouali et al. [17] reported other results, indicating that the methanol extract had greater antioxidant effects (IC_50_ = 268.02 ± 5.6 µg/mL) than the ethanolic (IC_50_ = 504.26 ± 17.53 µg/mL) and aqueous (IC_50_ = 528.3 ± 29.4 µg/mL) extracts. Other studies mentioned that the tepals exhibited the highest DPPH radical scavenging activity with an IC_50_ of 80.73 µg/mL, and the effective metal chelation abilities for Fe^2+^ and Cu^2+^ [64]. This demonstrated that the tepals have a significant capacity to reduce, or even eliminate, the radicals that build up in biological systems and whose accumulation contributes to a variety of pathological conditions, including cancer, inflammation, and neurodegenerative disorders [65].

### 3.4. Antimicrobial Potential

The antimicrobial activity of the saffron extracts was assessed against two Gram-negative microorganisms (*E. coli* and *P. aeruginosa*), two Gram-positive bacteria (*E. faecalis* and *B. cereus*), and two yeast strains of *C. albicans*, using the agar disc diffusion method. The results are displayed in Table 5. ASE and ACE showed the highest inhibition diameter against *P. aeruginosa* and *B. cereus* (16 mm and 15.08 mm) and (15 mm and 15 mm), respectively. These extracts also presented an antifungal effect on *C. albicans* CIP. The methanol extract showed antimicrobial activity against all the tested microorganisms. It presented the minimum inhibitory effect on *C. albicans* CIP (10.16 mm), and the maximum on *P. aeruginosa* (15 mm). The butanol and acetone extracts were not active against *C. albicans* CIP, whereas the ethyl acetate extract did not exert antimicrobial effects on *C. albicans* ATCC. These extracts exhibited the greatest bioactivity against *B. cereus* (13.08 mm), *E. faecalis* (13 mm), and *C. albicans* CIP (14.08 mm), respectively.

The extracts exhibited different antimicrobial activities against the tested bacteria and fungi, with MICs ranging from 12.5 to 64 mg/mL, whereas the MBC values ranged from approximately 25 to 186 mg/mL for antibacterial and antifungal activities (Table 6). The variability of the antimicrobial activity of extracts could be explained by the different biochemical profiles they possess, both in qualitative and quantitative terms (e.g., phenolics), as well as their relative biological properties [66]. If the MBC/MIC ratio is ≤4, the effect of the plant extract may be defined as bactericidal; in contrast, if the ratio is >4, the bioactivity is considered bacteriostatic [67]. The most abundant antimicrobial extract was the aqueous successive extract, which had the lowest MIC (12.5 mg/mL) against *B. cereus*. In addition, this extract and aqueous crude extract also exhibited bactericidal effects against *P. aeruginosa* and *B. cereus* (with MBC/MIC = 2 for both) and fungicidal ones against *C. albicans* CIP (MBC/MIC = 2 and MBC/MIC = 4, respectively). The methanol extract exhibited bactericidal potential against *E. faecalis* (MBC/MIC = 2), *B. cereus* (MBC/MIC = 2), and *P. aeruginosa* (MBC/MIC = 4) and a bacteriostatic effect against *E. coli* (MBC/MIC > 4); these results are in agreement with those reported in Jafari-Sales & Pashazadeh [68]. Indeed, these authors have reported that the methanolic extract of saffron tepals had bactericidal effects on *B. cereus, E. coli* and *P. aeruginosa* (MBC/MIC = 2 for all pathogens), with MIC values of 12.5, 50, and 100 mg/mL, respectively. Both the butanol and acetone extracts were bactericidal against *E. faecalis* and *B. cereus*. On the other hand, the ethyl acetate and aqueous extracts had fungicidal effects on *C. albicans* CIP, with MBC/MIC ratios of 100/50 and 50/25, respectively. Lachguer et al. [50] have shown that diethyl ether extract from the *C. sativus* flower was bioactive against *S. aureus*, with MIC and MBC values of 12.5 and 25 mg/mL, respectively. Other scholars have reported a lower antimicrobial activity of saffron tepals compared to that documented in the present study [68]. The main differences observed in these results can potentially be attributed to the biochemical composition of the saffron flowers, the extraction conditions, the solvents used, the sensitivity of the bacteria to the different extracts and bioactive molecules, and the methods employed to evaluate antibacterial activity. Maybe due to the composition of their cell wall, Gram-positive bacteria seemed to be more susceptible to the inhibitory effects of saffron tepal extracts than Gram-negative ones. In fact, as reported by Jafari-Sales and Pashazadeh [68] and Feizi et al. [69], Gram-negative bacteria have an outer membrane that is rich in lipopolysaccharides, which increases their resistance to antibacterial treatments. The antibacterial activity of saffron extracts against Gram-positive bacteria, such as *S. aureus* and *B. cereus*, is much stronger than that of Gram-negative *E. coli* also according to the research of Feizy et al. [70]. In addition, the contents of phenolics, flavonoids, hydrolysable tannins, and terpenes can be strongly associated with antimicrobial activity [71,72]. These compounds exhibit multiple mechanisms of action that contribute to both antibacterial and antiviral effects. These compounds can disrupt the integrity of bacterial membranes, leading to ion leakage and the loss of cellular homeostasis, as observed with terpenoids like carvacrol and thymol [73]. Additionally, they interact with extracellular proteins, thereby modulating membrane permeability and facilitating the release of intracellular constituents [74]. Furthermore, they may interfere with biofilm formation by disrupting bacterial adhesion and inhibiting viral fusion [75].

### 3.5. Result of ADMET

The ADMET (Absorption, Distribution, Metabolism, Excretion, and Toxicity) profile of the major phytochemical compounds in the sample was analyzed to assess their pharmacokinetic properties (Table 7). Molar refractivity values varied among the compounds, with 4-Hydroxybenzoic acid (35.42) showing the lowest value, while Petunidin 3-glucoside (120.64) exhibited the highest. Regarding total polar surface area (TPSA), Pelargonidin 3-glucoside (173.21 Å^2^) and Petunidin 3-glucoside (202.67 Å^2^) had the highest TPSA values, whereas 4-Hydroxybenzoic acid (57.53 Å^2^) and Resveratrol (60.69 Å^2^) showed the lowest.

The consensus log Po/w values indicated that Resveratrol (2.48) was the most lipophilic compound, while Petunidin 3-glucoside (−1.66) was the least. In terms of gastrointestinal (GI) absorption, Gallic acid, Epicatechin, 4-Hydroxybenzoic acid, Resveratrol, and Pelargonidin demonstrated high absorption, whereas Chlorogenic acid, Pelargonidin 3-glucoside, and Petunidin 3-glucoside had low absorption levels.

**Figure 3:**
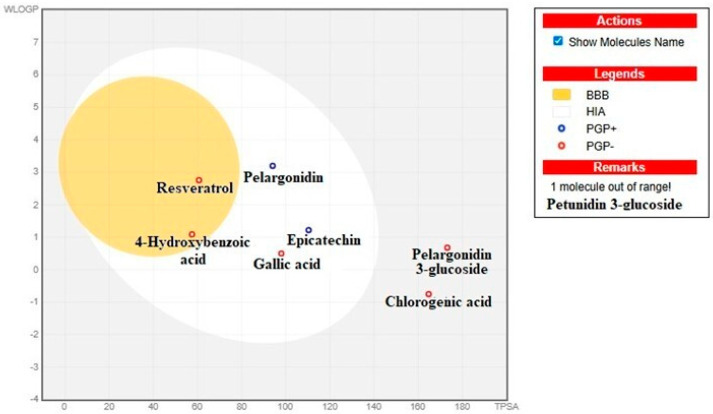
BBB and HIA predictions of phytochemical compounds.

Only 4-Hydroxybenzoic acid and Resveratrol exhibited blood-brain barrier permeability, while the other compounds did not seem to be able to cross the barrier. P-glycoprotein (P-gp) substrate analysis revealed that Epicatechin and Pelargonidin function as P-gp substrates (Figure 3). Cytochrome P450 enzyme inhibition analysis, instead, indicated that Resveratrol and Pelargonidin inhibited CYP1A2, while Resveratrol was also an inhibitor of CYP2C9. Additionally, Pelargonidin inhibited CYP2D6, and Gallic acid and Resveratrol inhibited CYP3A4. In terms of skin permeability (Log Kp) values, Resveratrol (−5.47 cm/s) showed the highest effect, whereas Petunidin 3-glucoside (−8.79 cm/s) the lowest. The bioavailability score analysis revealed that 4-Hydroxybenzoic acid (0.85) had the highest values, while Chlorogenic acid (0.11) and Petunidin 3-glucoside (0.17) the lowest ones. According to the synthetic accessibility (SA) index, 4-hydroxybenzoic acid (1.00) was identified as the most accessible compound for synthesis. In contrast, pelargonidin 3-glucoside (5.23) and petunidin 3-glucoside (5.46) exhibited greater structural complexity, rendering them less accessible for synthetic production. These findings provide a comprehensive overview of the pharmacokinetic characteristics, bioavailability potential, and metabolic profiles of the analyzed phytochemicals, offering a foundation for further biopharmaceutical and pharmacological research.

### 3.6. Molecular Docking Analysis of Binding Interactions and Inhibitory Potential

Molecular docking simulations were conducted to evaluate the interactions of selected phytochemical compounds with 8ACR (*P. aeruginosa* aminopeptidase), 2NRK (*E. faecalis* GrpB protein), 2NZF (*B. cereus* beta-lactamase II), and 3QNE (*C. albicans* seryl-tRNA synthetase). The binding affinity of each compound was assessed based on binding energy, ligand efficiency (LE), fit quality (FQ), estimated inhibition constant (Ki), and pIC_50_ values (Table 8).

Gallic acid exhibited the highest binding affinity with 8ACR (−6.8 kcal/mol, pIC_50_ = 4.857), supported by a ligand efficiency (LE) of 0.378, fit quality (FQ) of 0.598, and an estimated inhibition constant (Ki) of 10.365 μM. Its interactions with the other target proteins were 3QNE (−6.4 kcal/mol), 2NZF (−6.1 kcal/mol), and 2NRK (−5.9 kcal/mol), indicating moderate binding stability across multiple targets.

Epicatechin showed the strongest binding among all tested compounds, particularly with 8ACR (−9.5 kcal/mol, pIC_50_ = 6.786). Its interactions with 2NRK (−7.7 kcal/mol, pIC_50_ = 5.500) and 3QNE (−8.4 kcal/mol, pIC_50_ = 6.000) were also significant, while its binding to 2NZF (−6.7 kcal/mol, pIC_50_ = 4.786) was comparatively weaker. The high FQ values (0.859, 0.759, and 0.690) suggest that epicatechin adopts stable binding conformations within these target proteins.

4-Hydroxybenzoic acid exhibited its highest binding affinity toward 8ACR (−7.0 kcal/mol, pIC_50_ = 5.000), whereas its interactions with 2NRK (−5.7 kcal/mol), 2NZF (−5.5 kcal/mol), and 3QNE (−5.9 kcal/mol) were weaker, as indicated by their respective higher Ki values (66.354 μM, 92.997 μM, and 47.344 μM).

Chlorogenic acid demonstrated strong binding interactions with 3QNE and 2NRK (−8.5 kcal/mol, pIC_50_ = 6.071), followed by 8ACR (−8.4 kcal/mol, pIC_50_ = 6.000). The weakest interaction was observed with 2NZF (−6.9 kcal/mol, pIC50 = 4.929), indicating a lower affinity for this target.

Resveratrol exhibited the strongest interaction with 8ACR (−8.3 kcal/mol, pIC_50_ = 5.929), while its binding affinity to 2NRK (−7.5 kcal/mol, pIC_50_ = 5.357) and 3QNE (−7.7 kcal/mol, pIC_50_ = 5.500) was also notable. However, it showed a comparatively lower binding affinity for 2NZF (−6.2 kcal/mol, pIC_50_ = 4.429).

Pelargonidin displayed a strong binding affinity for 8ACR (−8.9 kcal/mol, pIC_50_ = 6.357), followed by 3QNE (−7.6 kcal/mol, pIC_50_ = 5.429) and 2NRK (−7.5 kcal/mol, pIC_50_ = 5.357). Its binding interaction with 2NZF (−6.8 kcal/mol, pIC_50_ = 4.857) was relatively weaker.

Pelargonidin 3-glucoside exhibited high binding stability with 3QNE (−8.7 kcal/mol, pIC_50_ = 6.214), while also showing strong interactions with 8ACR (−8.5 kcal/mol, pIC_50_ = 6.071) and 2NRK (−8.0 kcal/mol, pIC_50_ = 5.714). The lowest binding affinity was observed for 2NZF (−6.7 kcal/mol, pIC_50_ = 4.786).

Petunidin 3-glucoside displayed the highest binding energy among all compounds with 3QNE (−9.5 kcal/mol, pIC_50_ = 6.786), followed by strong interactions with 8ACR (−8.5 kcal/mol, pIC_50_ = 6.071) and 2NRK (−8.2 kcal/mol, pIC_50_ = 5.857). However, it showed a lower affinity for 2NZF (−6.9 kcal/mol, pIC_50_ = 4.929).

The results indicate that Epicatechin, Pelargonidin, Chlorogenic acid, and Petunidin 3-glucoside exhibited the highest binding affinities across multiple target proteins, as reflected by their low binding energy and high pIC_50_ values. Conversely, 4-Hydroxybenzoic acid and Gallic acid showed relatively weaker binding interactions. Among the target proteins, 3QNE and 8ACR demonstrated the strongest interactions with the tested compounds, suggesting a higher affinity for these phytochemicals.

A more detailed analysis provided insights into the binding mechanisms of these compounds through different interaction types, including hydrogen bonding, π-π stacking, π-alkyl interactions, π-cation interactions, and hydrophobic interactions (Supplemental Material—Table S1).

**Figure 4:**
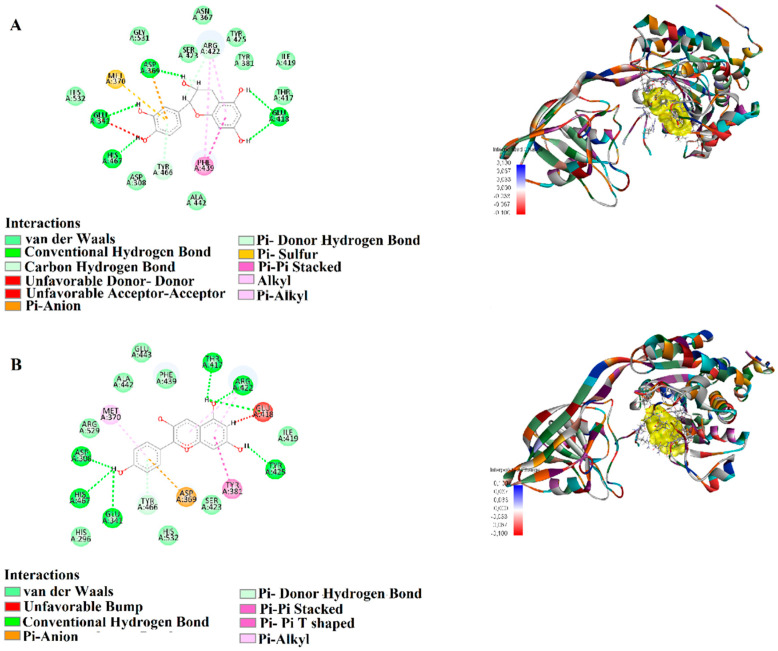
Molecular docking analysis of 2D and 3D interactions of (**A**) Epicatechin and (**B**) Pelargonidin with their respective target 8ACR.

Epicatechin exhibited strong binding stability with *P. aeruginosa* 8ACR, forming conventional hydrogen bonds (1.72–2.38 Å) with critical residues such as HIS467, ASP369, GLU418, GLU341, and ARG422. Additionally, a π-anion interaction (3.41 Å) with ASP369, a π-donor hydrogen bond (2.92 Å) with TYR466, and a π-π stacking interaction (4.01 Å) with PHE439 were observed. Hydrophobic interactions were identified with ARG422 and MET370 through π-alkyl interactions (4.18–5.23 Å), suggesting strong and stable binding to the target protein (Figure 4A). Pelargonidin also showed strong interaction with 8ACR, forming hydrogen bonds (1.72–2.69 Å) with ARG422, TYR425, THR417, GLU418, ASP308, and HIS467. Furthermore, a π-anion interaction (3.69 Å) with ASP369, π-π stacking interactions (5.34–5.67 Å) with TYR466 and TYR381, and π-alkyl interactions (3.94–5.23 Å) with ARG422 and MET370 were recorded (Figure 4B). These findings suggest that Pelargonidin exhibits high affinity and stability when bound to this target protein (Figure 4).

In recent years, the rise of multidrug-resistant bacteria has posed a serious threat to public health. *P. aeruginosa*, a Gram-negative bacterium frequently associated with hospital-acquired infections, is recognized as an opportunistic pathogen responsible for nosocomial infections [76]. In this context, molecular docking approaches are employed to identify drug-binding sites for the design of potent inhibitors targeting *P. aeruginosa* membrane proteins. In our study, docking analyses revealed that Epicatechin, 4-Hydroxybenzoic acid, Chlorogenic acid, Resveratrol, Pelargonidin, Pelargonidin 3-glucoside, and Petunidin 3-glucoside exhibited high binding affinities for 8ACR. These findings suggest that these compounds represent promising candidates for future pharmacological studies against *P. aeruginosa*. For example, Epicatechin, as the compound binding with the highest affinity, should not directly kill the bacteria; however, it would disrupt the function of aminopeptidase, thereby impairing biofilm formation and nutrient recycling, which in turn reduces bacterial growth and survival [77]. Thus, this study provides a valuable roadmap for designing new drugs to target this formidable pathogen amid increasing antibiotic resistance and highlights its significance in combating infectious diseases.

**Figure 5:**
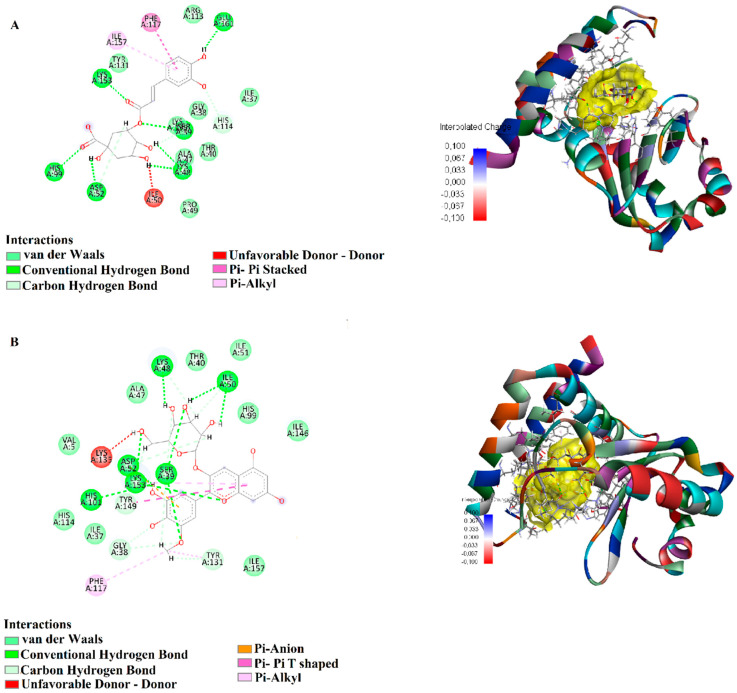
Molecular docking analysis of 2D and 3D interactions of (**A**) Chlorogenic acid and (**B**) Petunidin 3-glucoside with their respective target 2NRK.

Chlorogenic acid, when docked with *E. faecalis* 2NRK, formed hydrogen bonds (1.66–2.05 Å) with SER39, HIS99, LYS153, GLU161, and ASP52. Additionally, a π-π stacking interaction (4.00 Å) with PHE117 and a π-alkyl interaction (5.42 Å) with ILE157 were detected, highlighting stable interactions with this protein target (Figure 5A). Petunidin 3-glucoside, in its interaction with 2NRK, formed hydrogen bonds (2.05–2.85 Å) with residues such as SER39, LYS153, LYS48, ILE50, ASP52, and HIS101. Carbon-hydrogen bonds were also observed with GLY38 and TYR131 (2.06–2.76 Å). Moreover, π-anion interactions (3.78 Å) with ASP52 and π-π T-shaped interactions (4.87 Å) with TYR149 were identified, along with π-alkyl interactions (4.56–5.28 Å) with PHE117, TYR131, and LYS153, indicating a high binding affinity for this protein (Figure 5B).

In summary, Chlorogenic acid exhibited the highest binding affinity for 2NRK, showing strong interactions with the target protein, as supported by key hydrophobic interactions and hydrogen bonds. Compounds such as Petunidin 3-glucoside and Pelargonidin 3-glucoside showed the next highest binding energies after chlorogenic acid, with multiple hydrophobic interactions and hydrogen bonds enhancing their binding stability. 4-Hydroxybenzoic acid and Gallic acid displayed, instead, lower binding energies and interaction profiles, indicating comparatively lower potential activity. The comprehensive interaction profiles of Chlorogenic acid, Petunidin 3-glucoside, and Pelargonidin 3-glucoside highlighted their potential as potent antimicrobial agents. These findings warrant further investigation into their development as novel therapeutic candidates against *E. faecalis*, which is a Gram-positive bacterium that exhibits multidrug resistance and is responsible for approximately 70% of all enterococcal infections [78].

**Figure 6:**
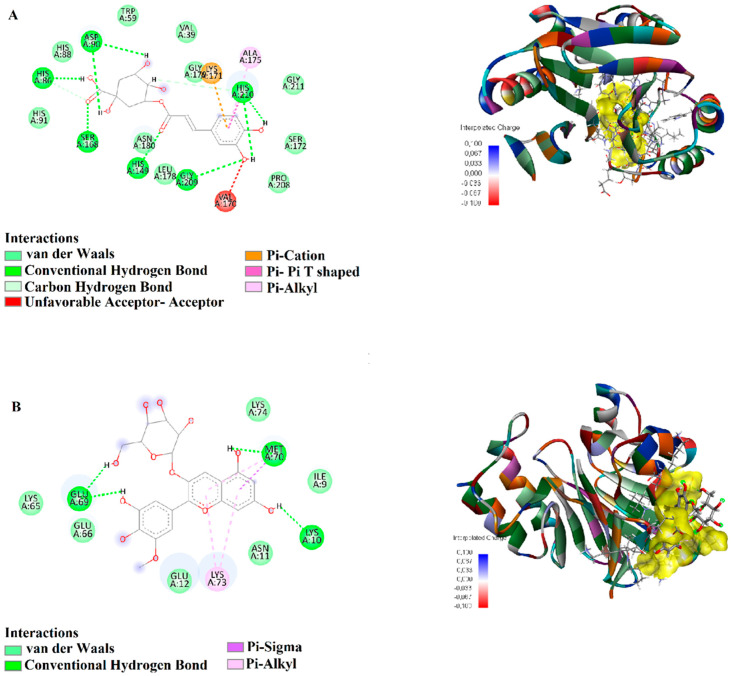
Molecular docking analysis of 2D and 3D interactions of (**A**) Chlorogenic acid and (**B**) Petunidin 3-glucoside, with their respective target 2NZF.

Chlorogenic acid, when docked with *B. cereus* 2NZF, established hydrogen bonds (1.52–2.86 Å) with HIS149, SER168, GLY209, HIS210, HIS86, and ASP90. Additional carbon-hydrogen bonds (2.34–2.96 Å) with HIS86 and HIS149 were observed, along with a π-cation interaction (3.22 Å) with LYS171 and a π-π T-shaped interaction (4.29 Å) with HIS210. These results suggest that chlorogenic acid binds effectively to 2NZF, contributing to its potential inhibitory effect (Figure 6A). Petunidin 3-glucoside, in its interaction with 2NZF, formed hydrogen bonds (1.62–2.16 Å) with MET70, LYS10, and GLU69. It also exhibited π-alkyl interactions (4.24–5.45 Å) with MET70 and LYS73, indicating a strong binding conformation (Figure 6B).

*B. cereus* is a Gram-positive, anaerobic, spore-forming bacterium associated with foodborne illness in humans. Vomiting and diarrhea are the primary symptoms of foodborne *B. cereus* infection, caused by emetic toxins and enterotoxins, respectively. Antibiotics, such as imipenem, vancomycin, chloramphenicol, gentamicin, and ciprofloxacin, are used for all susceptible *B. cereus* strains. However, these drugs may cause side effects in the host because the targeted proteins are not pathogen-specific and share similarities with human proteins [79]. Therefore, the present bioinformatic analysis focused on identifying potential novel metabolites targeting *B. cereus* 2NZF and inhibiting the pathogen’s function. Molecular docking revealed that Petunidin 3-glucoside, Pelargonidin 3-glucoside, Pelargonidin, Chlorogenic acid and Epicatechin exhibited high binding affinities with this protein. These results suggest that these phytochemicals could serve as potential inhibitors of *B. cereus*, representing promising pharmacological candidates for future studies.

**Figure 7:**
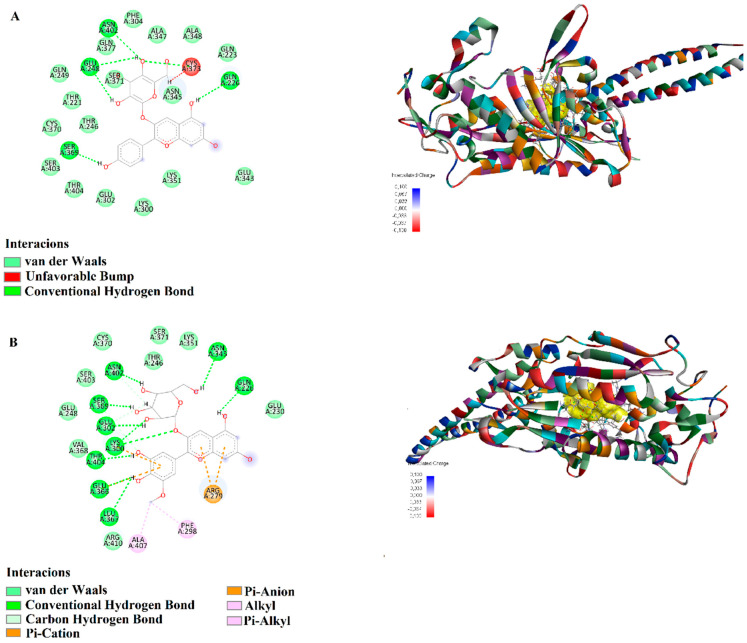
Molecular docking analysis of 2D and 3D interactions of (**A**) Pelargonidin 3-glucoside and (**B**) Petunidin 3-glucoside with their respective target 3QNE.

Pelargonidin 3-glucoside, in its interaction with *C. albicans* 3QNE, established hydrogen bonds (1.63–2.17 Å) with CYS373, ASN402, GLU248, SER369, and GLN226, demonstrating a strong and stable binding profile (Figure 7A). Petunidin 3-glucoside, when docked with 3QNE, formed hydrogen bonds (1.61–2.85 Å) with LYS300, ASN402, SER369, GLU302, GLN226, and ASN345. Additionally, π-cation interactions (3.37–3.71 Å) with ARG279 and LYS300, a π-anion interaction (4.35 Å) with GLU366, and a π-alkyl interaction (4.35 Å) with ALA407 were recorded, suggesting a high-affinity binding (Figure 7B).

Fluconazole and Voriconazole are widely used antifungal inhibitors for the treatment of fungal infections, including *C. albicans*. Unfortunately, these clinically used drugs exhibit significant side effects. Therefore, the development of safer and more effective therapeutic strategies has become increasingly necessary [80]. Interestingly, Epicatechin, Chlorogenic acid, Resveratrol, Pelargonidin, Pelargonidin 3-glucoside and Petunidin 3-glucoside were evaluated as potential antifungal substances, using in silico approaches. The results indicated that these compounds could serve as desirable agents for developing cost-effective and efficient antifungal therapeutics [81]. In detail, Epicatechin (−8.4 kcal/mol), Chlorogenic acid (−8.5 kcal/mol), Pelargonidin 3-glucoside (−8.7 kcal/mol), and Petunidin 3-glucoside (−9.5 kcal/mol) exhibited high binding affinities with the *C. albicans* seryl-tRNA synthetase (3QNE) protein. These findings suggest that these compounds may act as potent inhibitors of the 3QNE protein. Molecular docking revealed stable protein–ligand complexes for 3QNE, demonstrating their favorable binding tendencies and highlighting their potential as promising targets for drug development.

Overall, the docking results indicated that Epicatechin, Pelargonidin, Chlorogenic Acid, and Petunidin 3-glucoside exhibited strong binding interactions with the target proteins through multiple hydrogen bonds, π-π stacking, and hydrophobic interactions. Pelargonidin 3-glucoside and Petunidin 3-glucoside demonstrated powerful interactions with 3QNE, suggesting high stability and affinity. These two molecules may inhibit SerRS, a key enzyme in *C. albicans*, by disrupting its function, thereby affecting protein synthesis essential for fungal growth [82]. The structural analysis of SerRS suggests that targeting this enzyme could be a novel antifungal strategy, particularly given the increasing resistance to conventional antifungals [83]. In this study, Chlorogenic acid and Petunidin 3-glucoside exhibited strong binding with 2NRK and 2NZF, while Epicatechin and Pelargonidin had strong interactions with 8ACR. According to other studies, Chlorogenic acid and Petunidin 3-glucoside have demonstrated significant binding interactions with proteins. Indeed, Chlorogenic acid is known for its ability to form both covalent and non-covalent interactions with proteins, enhancing their functional properties [84]. Petunidin, a type of anthocyanidin, has shown strong binding capabilities with human serum albumin and the ability to induce notable conformational changes in DNA [85]. In contrast, Epicatechin and Pelargonidin documented strong interactions with the protein 8ACR, highlighting their potential in therapeutic applications against oxidative stress and drug resistance [86,87]. Anyway, while the antimicrobial assays and protein-ligand interaction predictions carried out in the present work constitute preliminary evidence, the data here described establish a robust framework for future validation (e.g., time-kill assay), offering critical leads for the targeted development of plant-derived antibiotics. Indeed, while the present study provides a robust characterization of the static interaction patterns between phytochemicals and microbial proteins, we acknowledge that docking-based observation represents a snapshot of the binding event. To further validate the temporal stability and the thermodynamic behaviour of the investigated complexes, future analyses need to be carried out, such as extensive molecular dynamics simulations (ideally for at least 100 ns), providing a deeper support to the structural data reported herein.

## 4. Conclusions

This study demonstrates that all *C. sativus* tepal extracts, here obtained using different types of solvents, are rich in phenols, flavonoids, flavonols, anthocyanins, and tannins. The concentration of these phytochemicals correlates directly with both antioxidant capacity and antibacterial activity, with the aqueous extracts exhibiting the most significant levels. Molecular docking simulations revealed strong binding interactions between target proteins and saffron phytochemicals, especially Epicatechin, Pelargonidin, Chlorogenic acid, and Petunidin 3-glucoside. Collectively, these findings highlight the promising potential of these molecules as antimicrobial or therapeutic agents, with versatile applications in enhancing food safety, developing nutraceuticals for oxidative stress-related diseases, and improving skin health in cosmetic formulations.

## Figures and Tables

**Table 1:** Yield and total content of polyphenolic compounds in the solvent successive extracts and the aqueous crude extract from saffron waste. Data are expressed as mg of standard equivalent per g of dry matter.

Extracts	Yield (%)	Total Phenolics (mg GAE/g DM)	Total Flavonoids (mg CE/g DM)	Total Flavonols (mg QE/g DM)	Hydrolysable Tannins (mg GAE/g DM)	Condensed Tannins (mg CE/g DM)
DSE	0.901	8.119 ± 0.02 ^a^	5.861 ± 0.904 ^a^	5.842 ± 0.103 ^a^	39.341 ± 0.165 ^a^	6.040 ± 1.284 ^a^
EASE	1.714	17.486 ± 0.026 ^b^	13.814 ± 0.821 ^b^	17.675 ± 1.191 ^b^	127.829 ± 4.750 ^b^	21.611 ± 1.703 ^b^
AcSE	2.061	23.293 ± 0.047 ^c^	19.866 ± 1.084 ^c^	31.536 ± 2.665 ^c^	125.061 ± 4.696 ^b^	38.541 ± 2.976 ^c^
BSE	6.278	40.332 ± 0.148 ^d^	25.280 ± 0.965 ^c^	45.398 ± 1.312 ^d^	160.75 ± 5.209 ^c^	50.552 ± 2.306 ^c; d^
MSE	28.394	60.405 ± 0.115 ^e^	30.995 ± 2.386 ^d^	57.823 ± 1.478 ^e^	250.733 ± 3.321 ^d^	41.083 ± 7.060 ^c^
ASE	26.455	70.532 ± 0.238 ^f^	39.283 ± 0.664 ^e^	50.967 ± 2.183 ^d; e^	160.924 ± 1.13 ^c^	56.814 ± 0.761 ^d; e^
ACE	62.433	75.152 ± 0.134 ^g^	47.647 ± 0.583 ^f^	52.862 ± 1.728 ^d; e^	120.83 ± 2.066 ^b^	66.665 ± 2.887 ^e^

Values not sharing the same letter are significantly different with *p* < 0.001 based on the Tukey test; DSE: dichloromethane successive extract; EASE: Ethyl acetate successive extract; AcSE: acetone successive extract; BSE: n-butanol successive extract; MSE: methanol successive extract; ASE: aqueous successive extract; ACE: aqueous crude extract.

**Table 2:** Concentrations of the phenolic compounds detected via HPLC-DAD analysis in the aqueous crude extract and the aqueous successive extract.

Detected Compound	ACE (ng/mg)	ASE (ng/mg)
4-Hydroxybenzoic acid	7.930 ± 0.396	9.621 ± 0.481
Gallic acid	3.694 ± 0.184	5.187 ± 0.311
Epicatechin	3.401 ± 0.204	3.624 ± 0.144
Chlorogenic acid	2.714 ± 0.023	3.327 ± 0.232
Resveratrol	2.617 ± 0.130	2.653 ± 0.106
Myricetin	1.844 ± 0.092	0.68 ± 0.047
Syringic acid	1.422 ± 0.071	1.3 ± 0.078
Vanillic acid	1.416 ± 0.070	N.D.
Quercetin-3-*O*-glucoside	0.763 ± 0.053	1.048 ± 0.062
Rosmarinic acid	0.730 ± 0.029	0.269 ± 0.013
Caffeic acid	0.637 ± 0.038	0.692 ± 0.041
Salicylic acid	0.614 ± 0.024	0.026 ± 0.001
5,7-Dimethoxycoumarin	0.550 ± 0.038	0.192 ± 0.007
*p*-Coumaric acid	0.549 ± 0.016	0.399 ± 0.019
Quercetin	0.459 ± 0.027	0.213 ± 0.010
Kaempferol	0.158 ± 0.006	0.068 ± 0.003
Genistein	0.144 ± 0.007	0.026 ± 0.001
1,1-Dimethylallyl caffeate	0.016 ± 0.000	0.018 ± 0.000

N.D.: not detected; ACE: aqueous crude extract; ASE: aqueous successive extract.

**Table 3:** Quantitation of anthocyanins.

Anthocyanin	Concentration (µg/mg)
Petunidin 3-glucoside	3.890 ± 0.233
Pelargonidin 3-glucoside	2.871 ± 0.143
Pelargonidin	2.018 ± 0.090
Cyanidin	1.624 ± 0.080
Malvidin	1.483 ± 0.096
Delphinidin	1.382 ± 0.041
Peonidin	1.127 ± 0.068

**Table 4:** Total antioxidant capacity (TAC) and DPPH scavenging radical assays.

Sample	TAC (mg AAE/g)	EC_50_ DPPH (mg/mL)
DSE	4.858 ± 0.623 ^a^	NA
EASE	20.253 ± 1.737 ^b^	6.579 ± 0.203 ^c^
AcSE	30.433 ± 1.523 ^c^	6.694 ± 0.176 ^c^
BSE	45.294 ± 3.826 ^d^	6.063 ± 0.419 ^c^
MSE	60.340 ± 1.791 ^e^	3.725 ± 0.482 ^b^
ASE	66.979 ± 0.82 ^e f^	2.646 ± 0.09 ^b^
ACE	72.437 ± 0.722 ^f^	2.669 ± 0.463 ^b^
Ascorbic acid		0.060 ± 0.001

Values not sharing the same letter are significantly different with *p* < 0.001 based on the Tukey test. NA: no activity; DSE: dichloromethane successive extract; EASE: ethyl acetate successive extract; AcSE: acetone successive extract; BSE: n-butanol successive extract; MSE: methanol successive extract; ASE: aqueous successive extract; ACE: aqueous crude extract.

**Table 5:** Bacterial and yeast inhibition zones (diameter in millimeters) obtained by treatment with the different extracts of *Crocus sativus* tepals.

Microorganism	DSE	EASE	AcSE	BSE	MSE	ASE	ACE	Standard Antibiotic
*E. coli*	NA	10 ± 0.90 ^b^	9.16 ± 0.87 ^b^	10 ± 0.25 ^b^	11.16 ± 0.87 ^b^	NA	NA	11 ± 1.41 ** (R)
*P. aeruginosa*	NA	10.08 ± 1.28 ^b^	11 ± 0.1 ^b^	11 ± 0.1 ^b^	15 ± 1.14 ^c^	16 ± 0.25 ^c^	15.08 ± 0.38 ^c^	NA
*E. faecalis*	NA	12 ± 1 ^b^	13 ± 0.1 ^b^	12 ± 0.1 ^b^	13.5 ± 0.1 ^b^	13 ± 0.1 ^b^	NA	8 ± 4.41 (R)
*B. cereus*	9 ± 0.1 ^a, b,^ *	7.5 ± 0.86 ^a^	12.08 ± 0.87 ^b, c^	13.08 ± 0.87 ^c^	13 ± 1 ^c^	15 ± 0.25 ^c^	15 ± 0.43 ^c^	NA
*C. albicans CIP*	9 ± 1 ^b^	14.08 ± 0.87 ^d^	NA	NA	10.16 ± 0.87 ^b, c^	12 ± 0.6 ^c, d^	13 ± 0.1 ^c; d^	39 ± 4.24 *** (S)
*C. albicans ATCC*	NA	NA	12 ± 0.1 ^b^	11.08 ± 1.28 ^b^	13.75 ± 0.86 ^b^	NA	NA	32.5 ± 0.70 (S)

Values not sharing the same letter are significantly different (*p* < 0.001) according to Tukey’s test; NA: no activity; Different letters indicate significant differences among saffron flower extracts and bacterial or yeast strains at *p* < 0.001, based on Tukey’s test; * Expressed as the size of the inhibition zones (mm) as an average of duplicates ± SD, including diameter of paper disk (6 mm); ** Ampicillin for bacteria (10 μg/disc); *** Nystatin for fungi (100 μg/disc); R: Microorganism classified as Resistant by CLSI criteria to the antimicrobial compound; S: Susceptible; DSE: dichloromethane successive extract; EASE: Ethyl acetate successive extract; AcSE: acetone successive extract; BSE: n-butanol successive extract; MSE: methanol successive extract; ASE: aqueous successive extract; ACE: Aqueous crude extract.

**Table 6:** Antimicrobial activity (MIC, MBC/MFC and ratio) of aqueous crude extracts and solvent successive extracts of *Crocus sativus* tepals.

Strains	EASE			AcSE			BSE			MSE			ASE			ACE		
	MIC	MBC or MFC	Ratio	MIC	MBC or MFC	Ratio	MIC	MBC or MFC	Ratio	MIC	MBC or MFC	Ratio	MIC	MBC or MFC	Ratio	MIC	MBC or MFC	Ratio
*E. coli*	/	/	/	/	/	/	/	/	/	64	>256	>4	/	/	/	/	/	/
*P. aeruginosa*	/	/	/	/	/	/	/	/	/	32	128	4	25	50	2	28.5	57	2
*E. faecalis*	25	100	4	62.5	125	2	46.5	186	4	32	64	2	25	100	4	/	/	/
*B. cereus*	/	/	/	31.25	62.5	2	23.25	46.5	2	16	32	2	12.5	25	2	14.25	28.5	2
*C. albicans CIP*	50	100	2	/	/	/	/	/	/	/	/	/	25	50	2	28.5	114	4
*C. albicans ATCC*	/	/	/	62.5	>125	>2	46.5	>93	>2	64	128	2	/	/	/	/	/	/

MIC: Minimum inhibitory concentration, MBC: Minimum bactericidal concentration, MFC: Minimum fungicidal concentration. MIC, MBC and MFC are expressed in mg/mL. Ratio = MBC/MIC for bacteria (*E. coli, P. aeruginosa, E. faecalis,* and *B. cereus*) or Ratio = MFC/MIC for fungi; EASE: Ethyl acetate successive extract; AcSE: acetone successive extract; BSE: n-butanol successive extract; MSE: methanol successive extract; ASE: aqueous successive extract; ACE: Aqueous crude extract.

**Table 7:** Acute toxicity profile values of major phytochemical components in *Crocus sativus* tepals.

ADMET	Gallic Acid	Epicatechin	4-Hydroxybenzoic Acid	Chlorogenic Acid	Resveratrol	Pelargonidin	Pelargonidin 3-glucoside	Petunidin 3-glucoside
Molar Refractivity	39.47	74.33	35.42	80.80	67.88	74.15	103.27	120.64
TPSA	97.99 Å^2^	110.38 Å^2^	57.53 Å^2^	164.75 Å^2^	60.69 Å^2^	94.06 Å^2^	173.21 Å^2^	202.67 Å^2^
Consensus Log *P*_o/w_	0.21	0.85	1.05	−0.39	2.48	0.93	−0.84	−1.66
GI absorption	High	High	High	Low	High	High	Low	Low
BBB permeant	No	No	Yes	No	Yes	No	No	No
P-gp substrate	No	Yes	No	No	No	Yes	No	No
CYP1A2 inhibitor	No	No	No	No	Yes	Yes	No	No
CYP2C19 inhibitor	No	No	No	No	No	No	No	No
CYP2C9 inhibitor	No	No	No	No	Yes	No	No	No
CYP2D6 inhibitor	No	No	No	No	No	Yes	No	No
CYP3A4 inhibitor	Yes	No	No	No	Yes	No	No	No
Log *K*_p_ (skin permeation)	−6.84 cm/s	−7.82 cm/s	−6.02 cm/s	−8.76 cm/s	−5.47 cm/s	−6.33 cm/s	−8.60 cm/s	−8.79 cm/s
Bioavailability Score	0.56	0.55	0.85	0.11	0.55	0.55	0.55	0.17
Synthetic accessibility	1.22	3.50	1.00	4.16	2.02	3.04	5.23	5.46

**Table 8:** Results of binding interactions of the compounds with target proteins.

		Binding Energy (kcal/mol)	Ligand Efficiency	Fit Quality (FQ)	Estimated Inhibition Constant {(Ki) (μM)}	pIC_50_
Gallic acid	8ACR	−6.8	0.378	0.598	10.365	4.857
	2NRK	−5.9	0.328	0.519	47.344	4.214
	2NZF	−6.1	0.339	0.537	33.781	4.357
	3QNE	−6.4	0.356	0.563	20.359	4.571
Epicatechin	8ACR	−9.5	0.271	0.859	0.109	6.786
	2NRK	−7.7	0.222	0.69	2.269	5.500
	2NZF	−6.7	0.191	0.606	12.271	4.786
	3QNE	−8.4	0.240	0.759	0.696	6.000
4-Hydroxybenzoic acid	8ACR	−7.0	0.438	0.599	7.395	5.000
	2NRK	−5.7	0.356	0.487	66.354	4.071
	2NZF	−5.5	0.344	0.470	92.997	3.929
	3QNE	−5.9	0.369	0.504	47.344	4.214
Chlorogenic acid	8ACR	−8.4	0.195	0.737	0.696	6.000
	2NRK	−8.5	0.198	0.746	0.588	6.071
	2NZF	−6.9	0.160	0.605	8.755	4.929
	3QNE	−8.5	0.198	0.746	0.588	6.071
Resveratrol	8ACR	−8.3	0.286	0.760	0.824	5.929
	2NRK	−7.5	0.259	0.687	3.180	5.357
	2NZF	−6.2	0.214	0.568	28.534	4.429
	3QNE	−7.7	0.266	0.705	2.269	5.500
Pelargonidin	8ACR	−8.9	0.287	0.812	0.299	6.357
	2NRK	−7.5	0.242	0.685	3.180	5.357
	2NZF	−6.8	0.219	0.621	10.365	4.857
	3QNE	−7.6	0.245	0.694	2.686	5.429
Pelargonidin 3-glucoside	8ACR	−8.5	0.163	0.716	0.588	6.071
	2NRK	−8.0	0.154	0.673	1.368	5.714
	2NZF	−6.7	0.129	0.564	12.271	4.786
	3QNE	−8.7	0.167	0.732	0.420	6.214
Petunidin 3-glucoside	8ACR	−8.5	0.147	0.695	0.588	6.071
	2NRK	−8.2	0.141	0.670	0.976	5.857
	2NZF	−6.9	0.119	0.564	8.755	4.929
	3QNE	−9.5	0.164	0.777	0.109	6.786

## Data Availability

The data that support the findings of this study are available from the corresponding author upon reasonable request.

## Supplementary Materials

The following supporting information can be downloaded at: https://www.mdpi.com/article/10.3390/biology15020197/s1, Table S1. Details of the interactions between the saffron phytochemicals and microbial proteins (8ACR: *P. aeruginosa* aminopeptidase; 2NRK: *E. faecalis* GrpB protein; 2NZF: *B. cereus* beta-lactamase II; 3QNE: *C. albicans* seryl-tRNA synthetase).
