# Supplementary material for: Biotechnological Potential of Algerian Saffron Floral Residues: Recycling Phytochemicals with Antimicrobial Activity
Source: Biology (Basel). 2026 Jan 21;15(2):197. doi: 10.3390/biology15020197 (PMC12837405; doi:10.3390/biology15020197)
Supplement: Supplementary file 1 [file biology-15-00197-s001.zip › biology-4069669-supplementary.pdf]

Supplemental Material - Table S1. Details of the interactions between the saffron phytochemicals and microbial proteins (8ACR: *P. aeruginosa* aminopeptidase; 2NRK: *E. faecalis* GrpB protein; 2NZF: *B. cereus* beta-lactamase II; 3QNE: *C. albicans* seryl-tRNA synthetase).

Ligand	Protein	Amino acids	Interacting	Distance
Epicatechin	8ACR	:[001:H1 - A:HIS467:NE2	Conventional Hydrogen Bond	1.72
		:[001:H6 - A:ASP369:O	Conventional Hydrogen Bond	2.36
		:[001:H10 - A:GLU418:OE2	Conventional Hydrogen Bond	1.91
		:[001:H12 - A:GLU418:O	Conventional Hydrogen Bond	1.99
		:[001:H14 - A:GLU341:OE1	Conventional Hydrogen Bond	2.38
		:[001:H4 - A:ARG422:O	Carbon Hydrogen Bond	2.53
		:[001:H5 - A:ASP369:OD2	Carbon Hydrogen Bond	2.82
		A:ASP369:OD2 - :[001	Pi-Anion	3.41
		A:TYR466:HH - :[001	Pi-Donor Hydrogen Bond	2.92
		A:MET370:SD - :[001	Pi-Sulfur	4.65
		A:PHE439 - :[001	Pi-Pi Stacked	4.01
		A:ARG422 - :[001	Alkyl	4.18
		A:PHE439 - :[001	Pi-Alkyl	4.47
		:[001 - A:ARG422	Pi-Alkyl	4.44
Pelargonidin	8ACR	A:ARG422:HH21 - :[001:O3	Conventional Hydrogen Bond	2.28
		:[001:H2 - A:TYR425:OH	Conventional Hydrogen Bond	1.93
		:[001:H4 - A:THR417:OG1	Conventional Hydrogen Bond	2.12
		:[001:H4 - A:GLU418:OE2	Conventional Hydrogen Bond	2.69
		:[001:H9 - A:ASP308:OD2	Conventional Hydrogen Bond	1.72
		:[001:H9 - A:GLU341:OE2	Conventional Hydrogen Bond	2.60
		:[001:H9 - A:HIS467:NE2	Conventional Hydrogen Bond	2.62
		A:ASP369:OD2 - :[001	Pi-Anion	3.69
		A:TYR466:HH - :[001	Pi-Donor Hydrogen Bond	2.70
		A:TYR381 - :[001	Pi-Pi Stacked	5.67
		A:TYR466 - :[001	Pi-Pi T-shaped	5.34
		:[001 - A:ARG422	Pi-Alkyl	4.29
		:[001 - A:ARG422	Pi-Alkyl	3.94
		:[001 - A:MET370	Pi-Alkyl	5.23
Chlorogenic acid	2NRK	A:SER39:HN - :[001:O3	Conventional Hydrogen Bond	2.05

		A:HIS99:HD1 - :[001:O8	Conventional Hydrogen Bond	2.05
		A:LYS153:HZ3 - :[001:O6	Conventional Hydrogen Bond	1.81
		:[001:H3 - A:LYS48:O	Conventional Hydrogen Bond	1.66
		:[001:H5 - A:LYS48:O	Conventional Hydrogen Bond	1.99
		:[001:H9 - A:GLU161:OE2	Conventional Hydrogen Bond	1.84
		:[001:H18 - A:ASP52:OD2	Conventional Hydrogen Bond	1.78
		A:SER39:HB2 - :[001:O2	Carbon Hydrogen Bond	2.54
		A:SER39:HB2 - :[001:O3	Carbon Hydrogen Bond	2.31
		A:HIS114:HA - :[001:O5	Carbon Hydrogen Bond	2.49
		:[001:H6 - :[001:O6	Carbon Hydrogen Bond	2.65
		:[001:H14 - A:ASP52:OD2	Carbon Hydrogen Bond	2.77
		A:PHE117 - :[001	Pi-Pi Stacked	4.00
		:[001 - A:ILE157	Pi-Alkyl	5.42
Petunidin 3-glucoside		2NRK		
		A:SER39:HN - :[001:O3	Conventional Hydrogen Bond	2.85
		A:SER39:HN - :[001:O9	Conventional Hydrogen Bond	2.15
		A:LYS153:HZ3 - :[001:O12	Conventional Hydrogen Bond	2.21
		A:LYS153:HZ2 - :[001:O6	Conventional Hydrogen Bond	2.79
		:[001:H1 - A:LYS48:O	Conventional Hydrogen Bond	2.12
		:[001:H2 - A:ILE50:O	Conventional Hydrogen Bond	2.07
		:[001:H3 - A:ILE50:O	Conventional Hydrogen Bond	2.00
		:[001:H13 - A:ASP52:OD1	Conventional Hydrogen Bond	2.05
		:[001:H13 - A:HIS101:NE2	Conventional Hydrogen Bond	2.54
		A:GLY38:HA1 - :[001:O10	Carbon Hydrogen Bond	2.36
		A:GLY38:HA2 - :[001:O9	Carbon Hydrogen Bond	2.36
		A:GLY38:HA2 - :[001:O10	Carbon Hydrogen Bond	2.15
		A:SER39:HB2 - :[001:O2	Carbon Hydrogen Bond	2.06
		:[001:H9 - A:TYR131:OH	Carbon Hydrogen Bond	2.32
		:[001:H11 - A:SER39:OG	Carbon Hydrogen Bond	2.76
		:[001:H17 - A:ASP52:OD2	Carbon Hydrogen Bond	2.40
		:[001:H18 - A:LYS48:O	Carbon Hydrogen Bond	2.55
		:[001:H18 - A:ILE50:O	Carbon Hydrogen Bond	2.71
		:[001:H22 - A:TYR149:OH	Carbon Hydrogen Bond	2.51
		A:ASP52:OD2 - :[001	Pi-Anion	3.78
		A:TYR149 - :[001	Pi-Pi T-shaped	4.87
		A:PHE117 - :[001:C18	Pi-Alkyl	4.56

		A:TYR131 - :[001:C18	Pi-Alkyl	5.28
		:[001 - A:LYS153	Pi-Alkyl	5.04
Chlorogenic acid	2NZF	A:HIS149:HD1 - :[001:O6	Conventional Hydrogen Bond	2.86
		A:SER168:HG - :[001:O8	Conventional Hydrogen Bond	1.52
		A:GLY209:HN - :[001:O5	Conventional Hydrogen Bond	2.85
		:[001:H3 - A:ASP90:OD2	Conventional Hydrogen Bond	2.67
		:[001:H9 - A:HIS210:O	Conventional Hydrogen Bond	1.68
		:[001:H10 - A:HIS210:O	Conventional Hydrogen Bond	2.01
		:[001:H17 - A:HIS86:NE2	Conventional Hydrogen Bond	1.92
		:[001:H18 - A:ASP90:OD2	Conventional Hydrogen Bond	2.41
		A:HIS86:HE1 - :[001:O8	Carbon Hydrogen Bond	2.96
		A:HIS149:HE1 - :[001:O6	Carbon Hydrogen Bond	2.34
		:[001:H6 - A:HIS210:NE2	Carbon Hydrogen Bond	2.76
		A:LYS171:NZ - :[001	Pi-Cation	3.22
		A:HIS210 - :[001	Pi-Pi T-shaped	4.29
		:[001 - A:LYS171	Pi-Alkyl	4.49
		:[001 - A:ALA175	Pi-Alkyl	4.15
Petunidin 3-glucoside	2NZF	:[001:H5 - A:MET70:O	Conventional Hydrogen Bond	2.14
		:[001:H6 - A:LYS10:O	Conventional Hydrogen Bond	1.80
		:[001:H13 - A:GLU69:OE1	Conventional Hydrogen Bond	1.62
		:[001:H20 - A:GLU69:OE1	Conventional Hydrogen Bond	2.16
		A:MET70:HG1 - :[001	Pi-Sigma	2.22
		:[001 - A:MET70	Pi-Alkyl	4.24
		:[001 - A:LYS73	Pi-Alkyl	4.64
		:[001 - A:LYS73	Pi-Alkyl	5.45
Pelargonidin 3-glucoside	3QNE	A:CYS373:HG - :[001:O3	Conventional Hydrogen Bond	2.08
		A:ASN402:HD21 - :[001:O3	Conventional Hydrogen Bond	2.74
		:[001:H4 - A:GLU248:OE2	Conventional Hydrogen Bond	2.12
		:[001:H6 - A:GLU248:OE1	Conventional Hydrogen Bond	2.01
		:[001:H9 - A:SER369:O	Conventional Hydrogen Bond	1.75
		:[001:H15 - A:GLN226:OE1	Conventional Hydrogen Bond	2.17
		:[001:H2 - :[001:O4	Carbon Hydrogen Bond	1.63

Petunidin 3-glucoside	3QNE	A:LYS300:HZ3 - :[001:O4	Conventional Hydrogen Bond	2.47
		A:LYS300:HZ3 - :[001:O5	Conventional Hydrogen Bond	2.85
		:[001:H1 - A:ASN402:OD1	Conventional Hydrogen Bond	1.61
		:[001:H2 - A:SER369:O	Conventional Hydrogen Bond	1.77
		:[001:H3 - A:GLU302:OE2	Conventional Hydrogen Bond	1.76
		:[001:H5 - A:GLN226:OE1	Conventional Hydrogen Bond	1.94
		:[001:H12 - A:GLU366:OE2	Conventional Hydrogen Bond	2.76
		:[001:H12 - A:LEU367:O	Conventional Hydrogen Bond	2.25
		:[001:H13 - A:THR404:OG1	Conventional Hydrogen Bond	1.64
		:[001:H20 - A:ASN345:OD1	Conventional Hydrogen Bond	2.28
		A:SER369:HB2 - :[001:O11	Carbon Hydrogen Bond	2.64
		:[001:H18 - A:GLU302:OE2	Carbon Hydrogen Bond	2.54
		:[001:H18 - A:ASN402:OD1	Carbon Hydrogen Bond	2.26
		A:ARG279:NH2 - :[001	Pi-Cation	3.71
		A:ARG279:NH2 - :[001	Pi-Cation	3.39
		A:LYS300:NZ - :[001	Pi-Cation	3.37
		A:GLU366:OE1 - :[001	Pi-Anion	4.35
		A:ALA407 - :[001:C18	Alkyl	4.35
		A:PHE298 - :[001:C18	Pi-Alkyl	4.50
